# Sacral acceleration can predict whole-body kinetics and stride kinematics across running speeds

**DOI:** 10.7717/peerj.11199

**Published:** 2021-04-12

**Authors:** Ryan S. Alcantara, Evan M. Day, Michael E. Hahn, Alena M. Grabowski

**Affiliations:** 1 Department of Integrative Physiology, University of Colorado Boulder, Boulder, CO, United States of America; 2 Department of Human Physiology, University of Oregon, Eugene, OR, United States of America

**Keywords:** Inertial measurement unit, Stress fracture, Ground reaction force, Injury, Machine learning, Biomechanics

## Abstract

**Background:**

Stress fractures are injuries caused by repetitive loading during activities such as running. The application of advanced analytical methods such as machine learning to data from multiple wearable sensors has allowed for predictions of biomechanical variables associated with running-related injuries like stress fractures. However, it is unclear if data from a single wearable sensor can accurately estimate variables that characterize external loading during running such as peak vertical ground reaction force (vGRF), vertical impulse, and ground contact time. Predicting these biomechanical variables with a single wearable sensor could allow researchers, clinicians, and coaches to longitudinally monitor biomechanical running-related injury risk factors without expensive force-measuring equipment.

**Purpose:**

We quantified the accuracy of applying quantile regression forest (QRF) and linear regression (LR) models to sacral-mounted accelerometer data to predict peak vGRF, vertical impulse, and ground contact time across a range of running speeds.

**Methods:**

Thirty-seven collegiate cross country runners (24 females, 13 males) ran on a force-measuring treadmill at 3.8–5.4 m/s while wearing an accelerometer clipped posteriorly to the waistband of their running shorts. We cross-validated QRF and LR models by training them on acceleration data, running speed, step frequency, and body mass as predictor variables. Trained models were then used to predict peak vGRF, vertical impulse, and contact time. We compared predicted values to those calculated from a force-measuring treadmill on a subset of data (*n* = 9) withheld during model training. We quantified prediction accuracy by calculating the root mean square error (RMSE) and mean absolute percentage error (MAPE).

**Results:**

The QRF model predicted peak vGRF with a RMSE of 0.150 body weights (BW) and MAPE of 4.27  ±  2.85%, predicted vertical impulse with a RMSE of 0.004 BW*s and MAPE of 0.80  ±  0.91%, and predicted contact time with a RMSE of 0.011 s and MAPE of 4.68  ±  3.00%. The LR model predicted peak vGRF with a RMSE of 0.139 BW and MAPE of 4.04  ±  2.57%, predicted vertical impulse with a RMSE of 0.002 BW*s and MAPE of 0.50  ±  0.42%, and predicted contact time with a RMSE of 0.008 s and MAPE of 3.50  ±  2.27%. There were no statistically significant differences between QRF and LR model prediction MAPE for peak vGRF (*p* = 0.549) or vertical impulse (*p* = 0.073), but the LR model’s MAPE for contact time was significantly lower than the QRF model’s MAPE (*p* = 0.0497).

**Conclusions:**

Our findings indicate that the QRF and LR models can accurately predict peak vGRF, vertical impulse, and contact time (MAPE < 5%) from a single sacral-mounted accelerometer across a range of running speeds. These findings may be beneficial for researchers, clinicians, or coaches seeking to monitor running-related injury risk factors without force-measuring equipment.

## Introduction

Stress fractures are a common running-related injury associated with the mechanical fatigue of bones in the pelvis, legs, and feet (Bennell et al., 1999) and affect 8–27% of runners worldwide (Brukner & Bennell, 1997; Brunet et al., 1990). Mechanical fatigue of bone tissue refers to the accumulation of damage and gradual decrease in strength resulting from repetitive loading (Edwards, 2018). Mechanical failure (stress fracture) will occur if repetitive loading continues without sufficient time for bone remodeling, and can require several months of decreased physical activity to fully recover (Matheson et al., 1987; Rizzone et al., 2017). Increases in bone loading peak magnitude and duration (total amount of time a bone is loaded) result in a decreased number of loading cycles until mechanical failure and influence the risk of stress fracture development (Edwards, 2018; Loundagin, Schmidt & Edwards, 2018). Although bone loading magnitude and duration have been measured in vivo, the invasiveness of this measurement limits the ability to monitor bone loading over weeks or months of running (Milgrom et al., 2007).

Instead of measuring bone loading directly, previous studies have estimated bone strains with finite element models (Edwards et al., 2009) and used external ground reaction forces to develop surrogate measures of internal bone loading (Kiernan et al., 2018; Matijevich et al., 2019). Although the vertical ground reaction force (vGRF) does not directly quantify the total force applied to the bones of the leg during running, research suggests that peak tibial bone loading (a combination of external reaction forces and internal muscle forces) occurs during midstance when the vGRF is greatest (Sasimontonkul, Bay & Pavol, 2007; Scott & Winter, 1990). Additionally, there is a moderate correlation between peak axial tibial compressive force (calculated as the sum of the 3-D GRF projected on the tibia and ankle torque divided by the Achilles tendon moment arm) and peak vGRF during running across a range of speeds and on uphill/downhill slopes (Matijevich et al., 2019). During running, peak vGRF is representative of the magnitude of external bone loading during stance phase and contact time is representative of external bone loading duration (Scott & Winter, 1990). Vertical impulse (integral of the vGRF with respect to contact time) considers both loading magnitude and duration and is representative of the total external loading with respect to time. For example, knee joint impulse has been investigated as a measure of total joint loading during running (Miller et al., 2014). However, peak vGRF, vertical impulse, and contact time are typically quantified using expensive and immobile force plates or force-measuring treadmills. Wearable devices provide a method for monitoring external loading variables longitudinally outside of a laboratory (Paquette et al., 2020), but the ability to simultaneously predict peak vGRF, vertical impulse, and contact time during running using a single wearable device and the accuracy of these predictions is not known. Predicting these biomechanical variables with a single wearable device could allow researchers, clinicians, and coaches to monitor external loading variables associated with bone loading magnitude and duration without the need of force-measuring equipment.

Inertial measurement units (IMUs) are wearable devices that contain an accelerometer, gyroscope, and magnetometer. These wearable devices can measure biomechanical variables in a variety of environments and have been used to measure limb segment accelerations during trail running (Giandolini et al., 2016), temporal variables (e.g., stride length, stride frequency) during marathons (Reenalda et al., 2016), and limb segment kinematics during an outdoor obstacle course (Vitali et al., 2019). Additionally, IMUs can be used to longitudinally monitor biomechanical variables that have been associated with running-related injuries. For example, IMUs have been used to measure peak tibial acceleration over the course of a marathon (Ruder et al., 2019) or estimate peak vGRF over several months of running (Kiernan et al., 2018); thus IMUs can be used to directly measure biomechanical variables (e.g., limb segment acceleration) and estimate biomechanical variables (e.g., whole-body kinetics). Although IMUs have been used to monitor runners and identify variables associated with prospective injuries (Davis, Bowser & Mullineaux, 2016), using IMUs to accurately estimate whole-body kinetic variables depends on sensor position (Tan et al., 2019), signal filtering (Day et al., 2021), number of sensors used (Karatsidis et al., 2016), and features included in predictive models (Neugebauer, Hawkins & Beckett, 2012).

Placing an IMU near the center of mass may allow for accurate estimations of the vGRF during running via acceleration data because the vGRF is the product of body mass and the vertical acceleration of the center of mass (accounting for gravity), assuming air resistance is neglected (Cavagna, Komarek & Mazzoleni, 1971). This estimate could then be used to calculate discrete variables such as peak vGRF, vertical impulse, and contact time. Such a physics-based approach would only require the use of one accelerometer. Using pelvis accelerations in this manner to estimate peak vGRF during treadmill running revealed moderate correlations with measures from gold standard force-measuring equipment across speeds (3.8–5.4 m/s; *r* = 0.64; Day et al., 2021) and moderate correlations when predicting the vGRF waveform during the stance phase of overground running (*r* = 0.50  ± 0.30; Gurchiek et al., 2017). Additionally, pelvis accelerations have been found to consistently overestimate (by ∼2.5 times) peak resultant COM acceleration (calculated by dividing the 3-D ground reaction force by body mass and subtracting gravitational acceleration) in runners across a range of speeds (2–5 m/s) (Nedergaard et al., 2017). However, applying more advanced analysis methods may provide more accurate predictions of whole-body kinetics and stride kinematics during running with a single sacral-mounted accelerometer.

Advanced data analysis techniques like machine learning have been used to estimate biomechanical variables based on IMU measurements during running (Derie et al., 2020; Johnson et al., 2021; Pogson et al., 2020; Wouda et al., 2018). However, training complex machine learning algorithms to predict vGRF data from wearable devices can result in limited model interpretability (the degree to which one can understand the cause of a machine learning model’s decision) (Miller, 2019). Similarly, the number of IMUs required to estimate a given biomechanical variable affects the financial cost of data collection and usability for coaches or clinicians interested in simultaneously collecting data on multiple individuals. One benefit of using IMUs instead of a force-measuring treadmill to measure whole-body kinetics and stride kinematics is the reduced financial cost of using IMUs (<$1000 vs. $100,000), but the financial benefit diminishes if multiple IMUs are needed to estimate biomechanical variables. Minimizing the number of IMUs required to estimate vGRF characteristics while balancing model interpretability and accuracy would ultimately improve the applicability of IMU-based research for researchers, clinicians, coaches, and athletes.

Supervised machine learning models like linear regressions and random forests have been used to model complex relationships between biomechanical measures and clinical outcomes (Backes et al., 2020; Halilaj et al., 2018). Linear regression (LR) models are often used to predict biomechanical outcomes because regression coefficients allow for interpretable predictions but LR models are generally limited to linear relationships between independent and dependent variables (Chambers, 1992). Conversely, quantile regression forests (QRF) are a type of ensemble random forest machine learning algorithm that can model linear or non-linear relationships between independent and dependent variables. However, a limitation of QRF algorithms is that they lack traditional regression coefficients that are present in LR models (Breiman, 2001; Meinshausen, 2006). Thus, QRF models can be more difficult to interpret despite potentially improved prediction accuracy (Marchese Robinson et al., 2017). Reporting the accuracy of multiple models applied to a dataset may illustrate this interpretability-accuracy tradeoff and present options that prioritize either interpretability or accuracy. Developing predictive models of biomechanical variables such as peak vGRF, vertical impulse, and contact time may improve the ability to quantify running-related injury risk by allowing coaches, clinicians, and researchers to use wearable devices to longitudinally monitor characteristics of loading outside of a laboratory setting.

The purpose of this study was to apply a QRF and LR model to data from a sacral-mounted accelerometer and determine the accuracy for predicting peak vGRF, vertical impulse, and ground contact time during running. Specifically, we used cross validation to train QRF and LR models to predict these biomechanical variables and compared them to gold standard measurements from a force-measuring treadmill. We hypothesized that the QRF model would have a lower mean absolute percent error (MAPE) than the LR model when predicting peak vGRF, vertical impulse, and contact time due to the model’s complexity and ability to account for potential non-linear changes across running speeds (Marchese Robinson et al., 2017; Nilsson & Thorstensson, 1989).

## Materials & Methods

### Participants

Thirty-seven National Collegiate Athletic Association (NCAA) Division I Cross Country runners (24 Female, 13 Male, 55.8  ± 9.7 kg, 170  ± 8 cm, 20  ± 2 years) from the University of Colorado Boulder and University of Oregon participated in this study. Participants were actively training and reported no musculoskeletal injuries at the time of data collection. The protocol was approved by the University of Colorado Boulder (protocol #: 17-0392) and University of Oregon (protocol #: 05162017.019) Institutional Review Boards and all participants provided written informed consent prior to data collection.

### Experimental design

Each participant ran on a level force-measuring treadmill (1000 Hz; Treadmetrix, Park City, UT or Bertec, Columbus, OH) for a series of consecutive 30-sec trials following a 5-minute self-paced warm up. Male participants ran at 3.8, 4.1, and 5.4 m/s while female participants ran at 3.8 and 4.9 m/s. We selected these speeds because they represent typical training run and race speeds for NCAA Division I Cross Country runners while maintaining a common speed between male and female participants. During all conditions, participants wore an accelerometer (500 Hz, 3 -axis ±16 g; IMeasureU, Centennial, CO) clipped posteriorly to the waistband of their running shorts near the sacrum as described in prior research (Fig. S1; Day et al., 2021). The accelerometer was positioned near the sacrum as vertical displacement of the sacrum is strongly correlated (*r* = 0.95) with vertical displacement of the center of mass during running (Napier et al., 2020).

### Data processing

Acceleration and vGRF data were collected during the final 10 s of each condition using separate data collection software and required temporal synchronization prior to data analysis. First, we down-sampled the vGRF data to match the sampling frequency of the accelerometer data (500 Hz). Then, we synchronized acceleration and vGRF data by having participants perform a countermovement jump on the stationary force-measuring treadmill at the beginning and end of the data collection session while simultaneously measuring acceleration and force. For each jump, we temporally aligned the estimated vGRF signal calculated from the acceleration data to the vGRF measured by the treadmill based on the cross correlation of their frequency content using a custom MATLAB (Mathworks, Natick, MA) script based on the work by Savorani, Tomasi & Engelsen (2013).

After time-synchronizing the data, we used a custom MATLAB (Mathworks, Natick, MA) script to filter and process the vGRF data measured by the instrumented treadmill and acceleration data measured by the accelerometer (Alcantara, 2019). We filtered vGRF data using a zero-lag 4th order low pass Butterworth filter with a 30 Hz cut-off. All stance phases during the final 10 s of each condition (approximately 30 steps) were used to calculate the mean peak vGRF, vertical impulse, contact time, and step frequency (number of initial ground contacts per second), discrete variables that were compared to each model’s predictions. Ground contact time was defined as the duration when the participant’s vGRF exceeded a threshold of 5% body weight (BW) (Day & Hahn, 2019).

The vertical acceleration data relative to the accelerometer’s local coordinate system were used for this analysis. We filtered acceleration data using a zero-lag 8th order low pass Butterworth filter with a 10 Hz cut-off, which isolates the frequency content corresponding to the vertical oscillation of the pelvis during running and the transient impact peak during early stance phase (Day et al., 2021). Ground contact time for accelerometer data was defined as the duration when the participant’s sacral acceleration exceeded a threshold of 0 m/s^2^ because the center of mass acceleration crosses 0 m/s^2^ at the start and end of the stance phase during running (Brabandere et al., 2018; Cavagna & Legramandi, 2015; Gaudino et al., 2013). We multiplied sacral acceleration by participant body mass to obtain an estimate of vGRF over the entire stance phase and normalized it to BW. Using this acceleration-based estimate of vGRF, we calculated peak vGRF, vertical impulse, and contact time for each condition. These discrete variables were used as inputs for the QRF and LR models.

### Data analysis

We compared the predictive accuracy of QRF and LR models using a train/test method. We chose to compare these models because LR models are generally interpretable due to the presence of regression coefficients but can be inaccurate when modeling nonlinear relationships and conversely, QRF models lack coefficients but can model nonlinear relationships. We partitioned the dataset into two subsets, with 28 runners (76% of enrolled participants: 9 Male, 19 Female; 65 total samples) used to train all models and 9 runners (24% of enrolled participants; 4 Male, 5 Female; 22 total samples) reserved to test model accuracy. Similar distributions of male and female runners were maintained in both subsets while ensuring that a runner’s data were not present in both subsets. This precaution was taken to provide conservative measures of model accuracy as model predictions were based on data from runners who were unknown to the model during training.

A QRF and LR model were constructed for predicting peak vGRF, vertical impulse, and contact time. In addition to the discrete variables calculated from the acceleration-based estimate, we included the runner’s body mass, step frequency, and running speed as predictor variables in both models, as these variables have been associated with changes in running biomechanics (Nagahara et al., 2018; Nilsson & Thorstensson, 1989). QRF models are a type of ensemble regression tree machine learning algorithm that can be used to predict a continuous numerical output instead of a classification label (Breiman, 2001; Meinshausen, 2006) and prior research suggests ensemble regression tree algorithms can be used to estimate vGRF characteristics using data from wearable devices (Derie et al., 2020). We used k-fold cross validation (5 folds) to train the models on the 76% subset and optimize the QRF parameters, minimizing for root mean squared error (RMSE) between model predictions and observed values. The QRF model predictions represent an ensemble of 500 regression tree predictions (Meinshausen, 2006). Then we used the trained models to make predictions based on the testing subset and quantified the error between model predictions and observed values from the force-measuring treadmill. RMSE, MAPE, and correlation coefficient (*r*) were calculated across the entire testing subset (22 predictions) and reported as prediction accuracy metrics. Paired t-tests (two-tailed, *α* = 0.05) were used to compare MAPE between models. Data analysis was performed in R (version 3.6.3) using custom scripts and packages (Arnold et al., 2019; Kuhn, 2020; Meinshausen, 2006; R Core Team, 2020; Wickham, 2009; Wickham & RStudio, 2020). For the LR models, the statistical significance level was set at *α* = 0.05 to determine coefficients that significantly contributed to model predictions during training.

## Results

Across all participants and conditions, mean  ± SD peak vGRF, vertical impulse, and contact time calculated from the force-measuring treadmill data were 2.94  ± 0.23 BW, 0.33  ± 0.02 BW*s, and 0.189  ± 0.017 s, respectively (Table 1). Mean ± SD QRF model predictions of peak vGRF, vertical impulse, and contact time were 2.95 ± 0.18 BW, 0.33 ± 0.02 BW*s, and 0.188 ± 0.017 s, respectively (Table 2). Mean ± SD LR model predictions of peak vGRF, vertical impulse, and contact time were 2.93 ± 0.18 BW, 0.33 ± 0.02 BW*s, and 0.189 ± 0.015 s, respectively (Table 2).

**Table 1:** Mean ± SD peak vertical ground reaction force (vGRF), vertical impulse, and contact time calculated from the ground reaction forces measured by the treadmill for all participants.

	Speed [m/s]	Peak vGRF [BW]	Vertical Impulse [BW*s]	Contact Time [s]
Females (*n* = 24)	3.8	2.79 ± 0.19	0.34 ± 0.02	0.201 ± 0.012
	4.8	2.94 ± 0.21	0.32 ± 0.02	0.175 ± 0.011
Males (*n* = 13)	3.8	2.94 ± 0.20	0.35 ± 0.01	0.204 ± 0.009
	4.1	3.00 ± 0.21	0.35 ± 0.01	0.196 ± 0.008
	5.4	3.14 ± 0.24	0.32 ± 0.01	0.168 ± 0.007

**Table 2:** Discrete variables calculated from the force-measuring treadmill data and predicted by the Quantile Regression Forest (QRF) or Linear Regression (LR) models for the testing subset of data. Mean ± SD peak vertical ground reaction force (vGRF), vertical impulse, and contact time for the nine participants in the testing subset of data.

**Peak vGRF [BW]**	**Speed [m/s]**	**Observed**	**QRF Prediction**	**LR Prediction**
**Females**	3.8	2.66 ± 0.18	2.78 ± 0.13	2.75 ± 0.05
(*n* = 5)	4.8	2.86 ± 0.15	2.88 ± 0.09	2.86 ± 0.05
**Males**	3.8	2.98 ± 0.08	2.98 ± 0.20	2.94 ± 0.13
(*n* = 4)	4.1	3.07 ± 0.10	3.02 ± 0.16	3.00 ± 0.15
	5.4	3.30 ± 0.20	3.14 ± 0.10	3.18 ± 0.16
Vertical Impulse [BW*s]				
**Females**	3.8	0.33 ± 0.02	0.33 ± 0.02	0.33 ± 0.02
(*n* = 5)	4.8	0.31 ± 0.02	0.31 ± 0.01	0.31 ± 0.02
**Males**	3.8	0.35 ± 0.01	0.35 ± 0.01	0.35 ± 0.01
(*n* = 4)	4.1	0.35 ± 0.01	0.35 ± 0.01	0.35 ± 0.01
	5.4	0.33 ± 0.01	0.33 ± 0.01	0.33 ± 0.01
**Contact Time [s]**				
**Females**	3.8	0.204 ± 0.014	0.198 ± 0.004	0.198 ± 0.008
(*n* = 5)	4.8	0.177 ± 0.012	0.167 ± 0.005	0.174 ± 0.008
**Males**	3.8	0.204 ± 0.009	0.203 ± 0.006	0.204 ± 0.005
(*n* = 4)	4.1	0.195 ± 0.007	0.201 ± 0.005	0.198 ± 0.005
	5.4	0.165 ± 0.007	0.173 ± 0.010	0.172 ± 0.002

**Figure 1:**
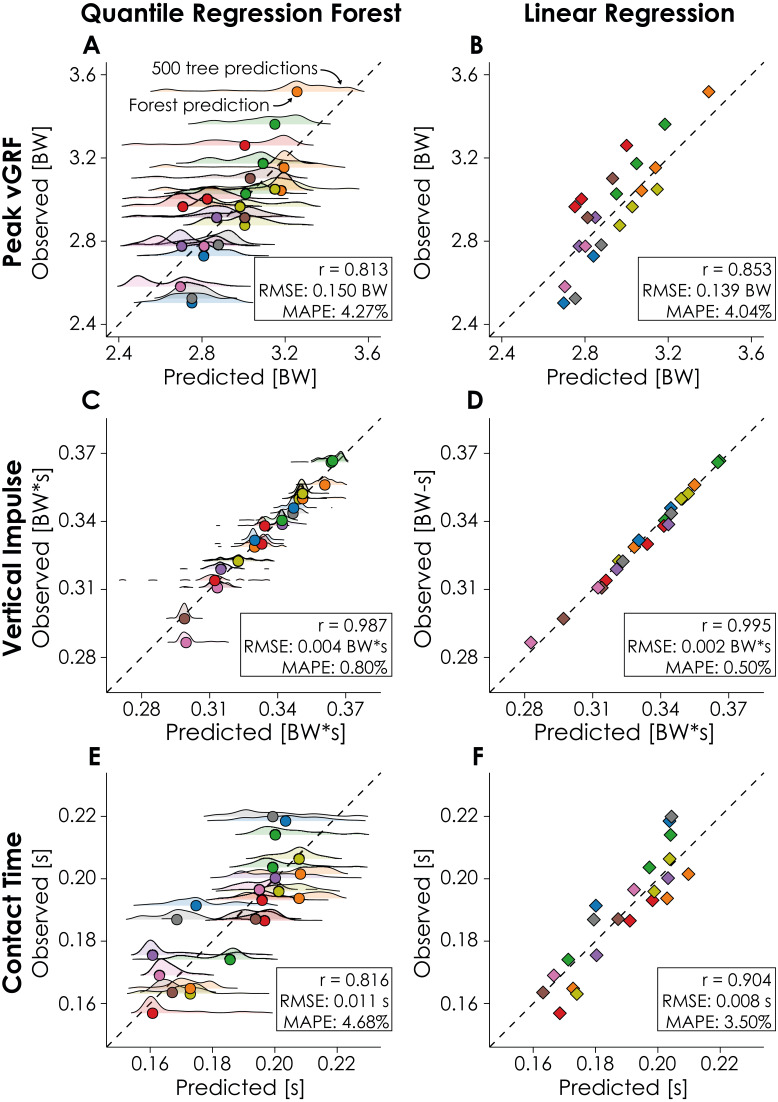
Quantile regression forest (QRF) and Linear regression (LR) model predictions. Model predictions (horizontal axes; QRF: circles, LR: diamonds) of peak vertical ground reaction force (vGRF), vertical impulse, and contact time are compared to the observed values (vertical axes) from the force-measuring treadmill. Dashed lines represent the line of identity, each point represents the value for a given condition-participant combination, and colors represent different participants in the testing subset (*n* = 9). Male participants completed three conditions and female participants completed two conditions. QRF model predictions are based on the predictions of 500 regression trees, with the distribution of tree predictions represented by the ridge plots.

Cross validation of the QRF model revealed that the optimal number of variables randomly sampled as candidates for each split in each regression tree was 2 when predicting peak vGRF and 3 when predicting vertical impulse or contact time. When applying the QRF model to the testing subset, model predictions of peak vGRF had a RMSE of 0.150 BW and MAPE ± SD of 4.27 ± 2.85%, predictions of vertical impulse had a RMSE of 0.004 BW*s and MAPE of 0.80 ± 0.91%, and predictions of contact time had a RMSE of 0.011 s and MAPE of 4.68 ± 3.00% (Fig. 1).

Cross validation of the LR model revealed that running speed, acceleration-based estimations, and step frequency contributed significantly to model predictions of peak vGRF (*p* = 0.000, *p* = 0.000, and *p* = 0.006 respectively; Table 3). Step frequency was the only predictor variable that significantly contributed to predictions of vertical impulse (*p* = 0.000; Table 3). Lastly, running speed, acceleration-based estimations, and body mass contributed significantly to predictions of contact time (*p* = 0.000, *p* = 0.019, and *p* = 0.000 respectively; Table 3). When applying the LR model to the testing subset, we found that model predictions of peak vGRF had a RMSE of 0.139 BW and MAPE of 4.04 ± 2.57%, predictions of vertical impulse had a RMSE of 0.002 BW*s and MAPE of 0.50 ± 0.42%, and predictions of contact time had a RMSE of 0.008 s and MAPE of 3.50 ± 2.27% (Fig. 1).

**Table 3:** Linear regression (LR) coefficients following cross validation on the training subset.

**Peak vGRF [BW]**	*y*= 2.23 + 0.15*Speed + 0.33* Accel-based Est. −0.34*Step Freq.		
	*B*	*SE*	*t*	*p*
Intercept	2.23	0.46	4.87	**0.000**
Speed [m/s]	0.15	0.03	4.42	**0.000**
Acceleration-based Estimate [BW]	0.33	0.05	6.54	**0.000**
Step Frequency [Hz]	−0.34	0.12	−2.84	**0.006**
Body Mass [kg]	0.001	0.003	0.50	0.621
Vertical Impulse [BW*s]	*y*= 0.69 − 0.10*Step Freq.			
	*B*	*SE*	*t*	*p*
Intercept	0.69	0.02	31.13	**0.000**
Speed [m/s]	−0.002	0.001	−1.73	0.089
Acceleration-based Estimate [BW*s]	−0.05	0.03	−1.79	0.079
Step Frequency [Hz]	−0.10	0.004	−25.95	**0.000**
Body Mass [kg]	0.00003	0.00009	0.32	0.752
**Contact Time [s]**	*y*= 0.230 − 0.019*Speed + 0.151*Accel-based Est. + 0.0007*Body Mass			
	*B*	*SE*	*t*	*p*
Intercept	0.230	0.033	6.88	**0.000**
Speed [m/s]	−0.019	0.002	−8.62	**0.000**
Acceleration-based Estimate [s]	0.151	0.063	2.42	**0.019**
Step Frequency [Hz]	−0.011	0.007	−1.51	0.135
Body Mass [kg]	0.0007	0.0002	4.05	**0.000**

**Notes.**

Unstandardized coefficients (*B*), coefficient standard errors (*SE*), *t* values (*t*), and *p* values (*p*) are listed for independent variables used to predict peak vertical ground reaction force (vGRF), vertical impulse, and contact time, where Accel-based Est. is the Acceleration-based estimate and Freq. is Frequency. Equations with statistically significant predictor variables are included. Bold *p* values indicate *p* < 0.05.

There were no statistically significant differences between the QRF and LR model MAPE when predicting peak vGRF (*p* = 0.549), or vertical impulse (*p* = 0.073). However, the LR model predicted contact time with significantly less error based on MAPE (*p* = 0. 0497), compared to the QRF model.

## Discussion

We developed QRF and LR models and quantified their accuracy when predicting peak vGRF, vertical impulse, and contact time from a sacral mounted IMU on data withheld during model training. We found that QRF predictions had a MAPE of 0.80–4.68% and the LR predictions had a MAPE of 0.50–4.04% for these biomechanical variables. Both models had the lowest MAPE when predicting vertical impulse, which may be due to the inclusion of step frequency in our models. For example, step frequency strongly contributed to the LR model prediction of vertical impulse (*B* =  − 0.10; *p* = 0.000; Table 3). This finding corroborates prior research that observed a strong negative correlation (*r* = −0.871 to −0.968 ) between step frequency and vertical impulse during overground sprinting (Nagahara et al., 2018). The QRF and LR models theoretically provide two levels of model accuracy and interpretability when predicting biomechanical variables with an accelerometer, but our data did not entirely support our hypothesis as both model types performed similarly when predicting peak vGRF and vertical impulse. Average differences in MAPE between the QRF and LR model predictions of peak vGRF and vertical impulse were ≤ 0.30% but when predicting contact time, the LR model had a significantly lower MAPE (3.50 ± 2.27%) than the QRF model (4.68 ± 3.00%; *p* = 0.0497). Additionally, the LR model predictions were more strongly correlated with the observed values from the treadmill data compared to the QRF model predictions (Fig. 1). These findings suggest that the LR model may more accurately describe the relationship between the predictor variables and contact time across the range of speeds tested (3.8–5.4 m/s; Table 2).

Prior research that has predicted biomechanical variables using wearable devices has utilized multiple IMUs or machine learning algorithms like artificial neural networks, which can be difficult to interpret despite low prediction errors (Johnson et al., 2021; Pogson et al., 2020; Wouda et al., 2018). This may ultimately limit the applicability of these prior findings because of the financial cost of using multiple IMUs or the computational requirements of applying the model. In the present study, we implemented a physics-based methodology of measuring acceleration at the sacrum from a single accelerometer clipped to a runner’s waistband. Our predictive models also required the runner’s body mass, speed, and step frequency, which can be measured using a scale, treadmill or GPS watch, and a stopwatch, respectively. We predicted biomechanical variables with a MAPE <5% by using a single accelerometer, which reduces the financial cost of measuring biomechanical variables yet maintains prediction accuracy comparable to that achieved using multiple wearable devices, kinematic data from 3-D motion capture, and artificial neural networks. For example, Wouda et al. (2018) predicted peak vGRF during running using three IMUs and an artificial neural network with an average RMSE of 0.38 BW and Komaris et al. (2019) achieved a mean error ∼0.10 BW when predicting peak vGRF using 3-D motion capture data and an artificial neural network. In contrast, our LR model predictions based on a single accelerometer achieved an RMSE of 0.139 BW for peak vGRF. Additionally, the RMSE of LR model predictions was smaller than the standard deviation of the peak vGRF, vertical impulse, and contact time values measured by the force-measuring treadmill (Table 1). We provided regression coefficients for the LR model (Table 3), which can be used to predict peak vGRF, vertical impulse, and contact time when provided with a runner’s speed, sacral acceleration, step frequency, and body mass. The LR model coefficients may be useful for coaches or clinicians interested in monitoring peak vGRF, vertical impulse, or contact time in runners over the course of a season or during injury rehabilitation without needing to apply a complex machine learning algorithm or use a force-measuring treadmill.

We predicted variables that characterize the magnitude and duration of external loading using wearable device data, and these data could be used to predict more complex surrogates of internal bone loading or identify stress fracture risk in runners via more accurate estimations of variables that affect cumulative external loading. For example, prior research has proposed surrogates of internal bone loading, such as peak axial tibial compression, that have a weak-moderate correlation with vertical impulse (r = -0.46 ± 0.40) and peak vGRF (*r* = 0.72 ± 0.42) across a range of speeds and slopes (Matijevich et al., 2019). We have shown that these external loading variables can be predicted with wearable devices. It may be possible to map wearable device data to peak axial tibial compression during running using similar techniques. However, it is unknown if peak axial tibial compression can be used to prospectively identify stress fracture risk despite the association between a bone’s load and risk of mechanical failure. If bone loading metrics can be identified as biomechanical risk factors, data collected by wearable devices could improve our understanding of stress fracture development in long distance runners as these data could be collected over the course of a run, competitive season, or several years in the environment experienced by runners daily and not only within a laboratory (Backes et al., 2020; Edwards, 2018; Ryan et al., 2020). Biomechanical risk factors could then be considered alongside other metrics such as bone mineral density or nutritional deficiencies when determining an individual’s stress fracture risk (Wright et al., 2015).

There are potential limitations of the present study that may limit the generalizability of our findings. There may be a tendency for the QRF and LR models to overestimate lower peak vGRF and contact times and underestimate higher peak vGRF and contact times (Figs. 1E and 1F). Variations in peak vGRF and contact time within a participant were due in part to changes in speed, so these biomechanical predictions may not generalize outside of the range of speeds tested (3.8–5.4 m/s). QRF model predictions may have benefitted from additional predictor variables as ensemble forests algorithms can be used to make predictions from hundreds or thousands of predictor variables (Breiman, 2001). However, we intentionally limited the number of predictor variables to increase model interpretability, likely at the cost of prediction accuracy. We collected data from level running and the prediction accuracy of the models may be affected by changes in slope, as uphill and downhill running affects kinematics and kinetics (Vernillo et al., 2020). The method we used to attach the accelerometer to the body may have introduced error in the acceleration data (Gurchiek et al., 2017). Specifically, the application of Newton’s second law of motion assumes acceleration is measured at the center of mass and not the sacrum. Additionally, we did not rotate the acceleration signal to be vertical in the global coordinate system, but instead used the vertical axis in the local coordinate system of the accelerometer, which may have rotated with changes in waistband position or pelvic orientation during running and influenced prediction accuracy (Tan et al., 2019). However, our decision to attach the accelerometer to the runner’s waistband and not rotate the vertical acceleration signal were in an effort to maintain the generalizability of results. Thus, our methodology presents conservative measures of model accuracy as using the global vertical acceleration from a device adhered directly to the skin would have likely improved signal quality. However, we observed predictions within 5% of peak vGRF, vertical impulse, and contact time calculated from gold standard force-measuring treadmill data across a range of speeds. The entirety of our participant population consisted of NCAA Division I Cross Country runners who may not be representative of the general recreational running population. Differences in running biomechanics have been observed when comparing runners of different skill levels or weekly running mileage (Boyer, Silvernail & Hamill, 2014; Cavanagh, Pollock & Landa, 1977; García-Pinillos et al., 2019; Mo et al., 2020) and these differences may affect model prediction accuracy when applied to other running subpopulations. However, by testing the QRF and LR models on data withheld during model training, we provided a measure of model accuracy when applied to the data of runners who were unknown to the models.

## Conclusions

We investigated the ability of quantile regression forest (QRF) and linear regression (LR) models to predict peak vertical ground reaction force, vertical impulse, and ground contact time in NCAA Division I Cross Country runners using sacral acceleration across a range of running speeds (3.8–5.4 m/s). Both models predicted these biomechanical variables on data withheld during model training with a mean absolute percent error (MAPE) <5%. Our data indicate that a sacral-mounted accelerometer can be used to predict peak vGRF (RMSE: ≤ 0.150 BW), vertical impulse (RMSE: ≤ 0.003 BW*s), and contact time (RMSE: ≤ 0.011 s) during running. We also provide LR model coefficients (Table 3), used to predict peak vGRF, vertical impulse, and contact time from sacral-mounted accelerometer data. Accurate longitudinal monitoring of these biomechanical variables may aid in the quantification of stress fracture risk in runners.

##  Supplemental Information

10.7717/peerj.11199/supp-1 Figure S1 Attachment method of the inertial measurement unit

## Additional Information and Declarations

### Data Availability

The following information was supplied regarding data availability: Data and code used to perform the analysis and generate the figures are available at GitHub: https://github.com/alcantarar/Sacral_Accel_Manuscript.
