# Supplementary figures and images for: Sacral acceleration can predict whole-body kinetics and stride kinematics across running speeds
Source: PeerJ. 2021 Apr 12;9:e11199. doi: 10.7717/peerj.11199 (PMC8048400; doi:10.7717/peerj.11199)

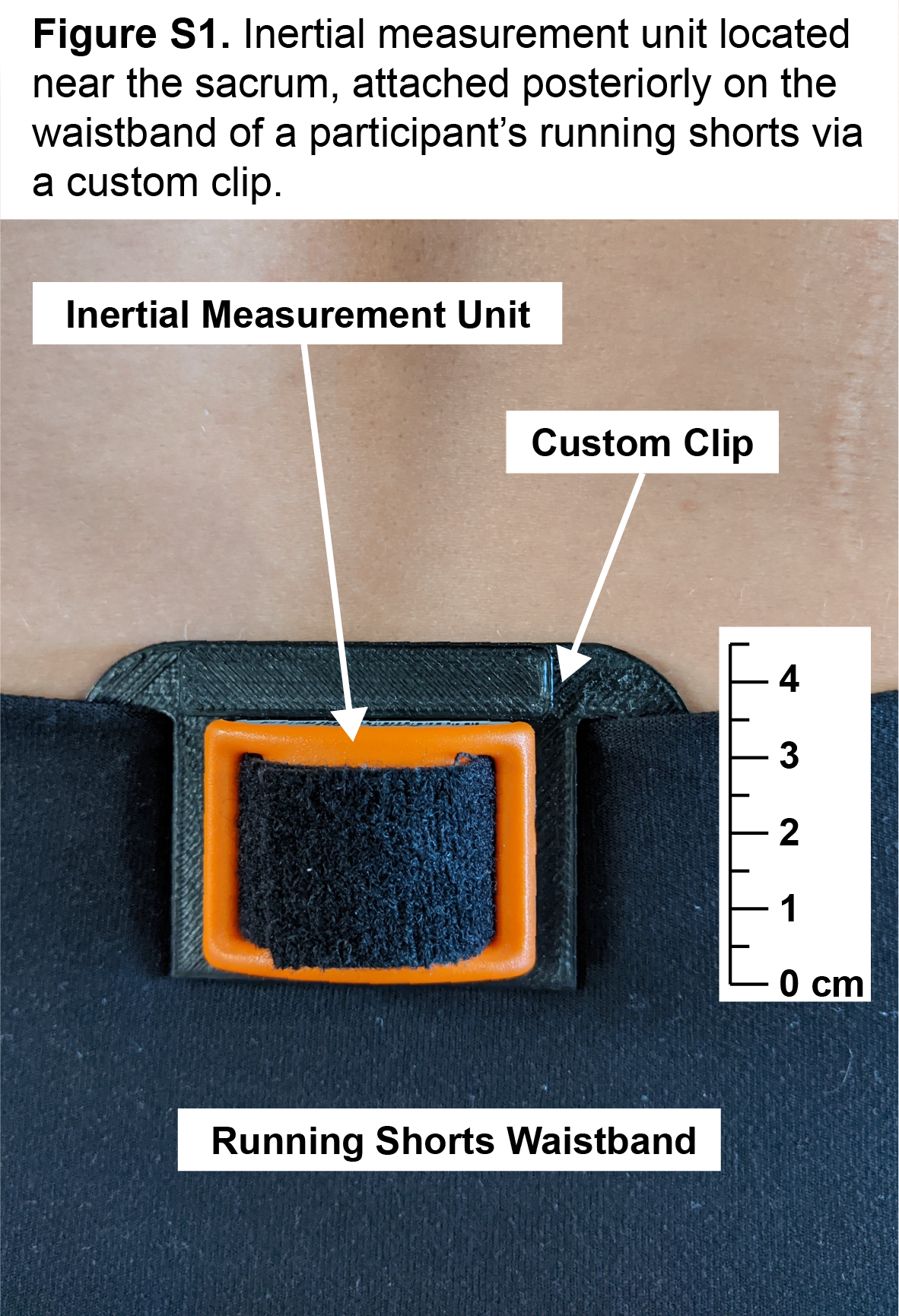

Supplement: Figure S1 [file peerj-09-11199-s001.png]
